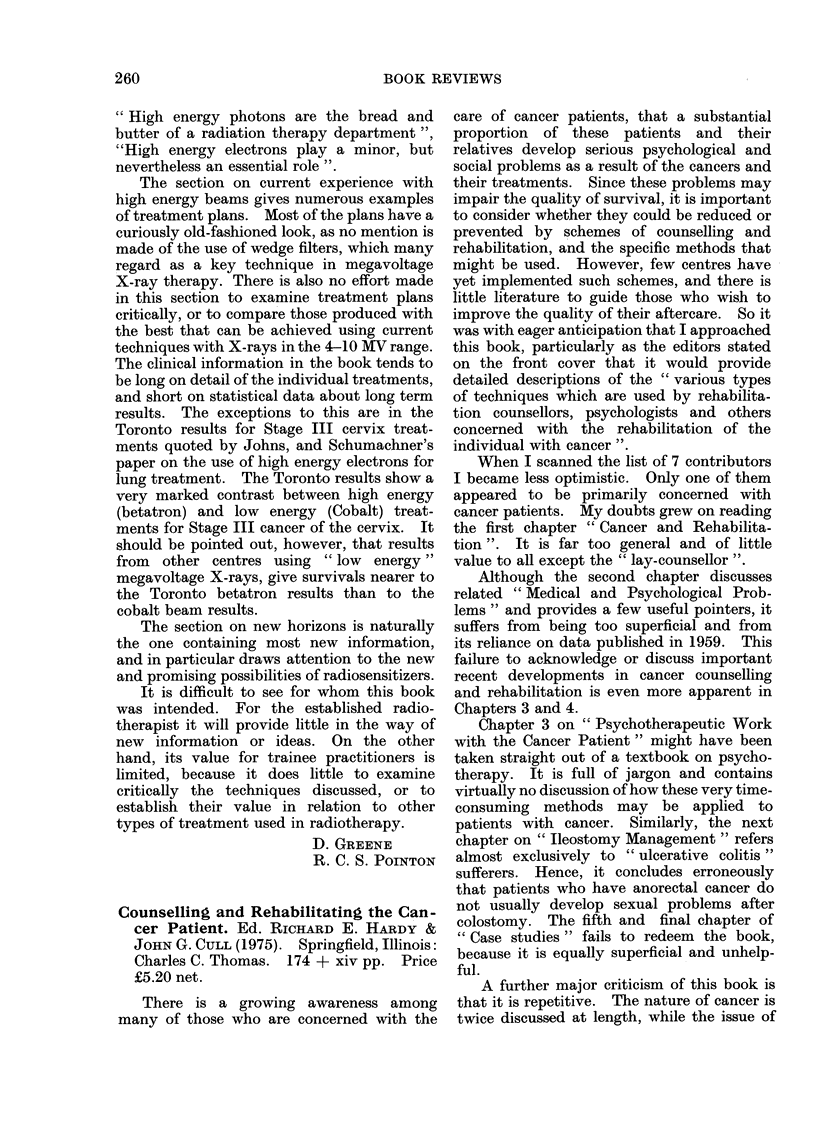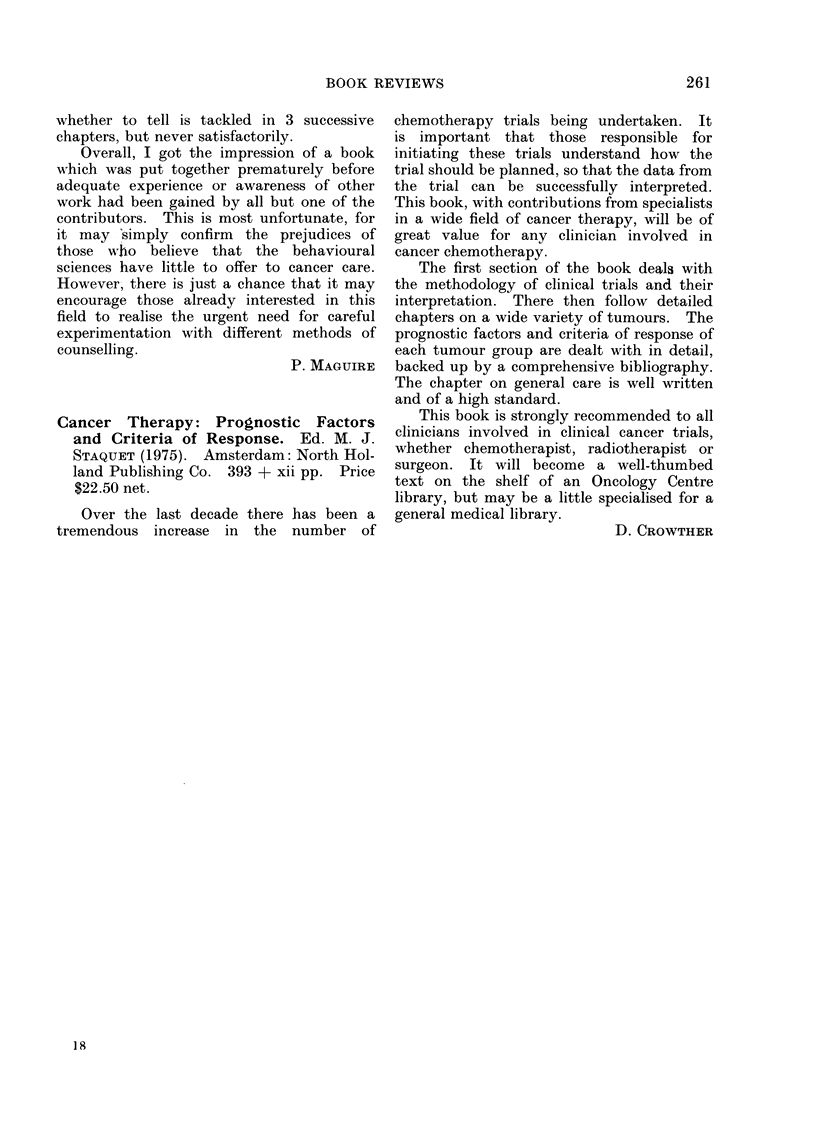# Counselling and Rehabilitating the Cancer Patient

**Published:** 1977-02

**Authors:** P. Maguire


**Counselling and Rehabilitating the Cancer Patient.** Ed. Richard E. Hardy & John G. Cull (1975). Springfield, Illinois: Charles C. Thomas. 174 + xiv pp. Price £5.20 net.

There is a growing awareness among many of those who are concerned with the care of cancer patients, that a substantial proportion of these patients and their relatives develop serious psychological and social problems as a result of the cancers and their treatments. Since these problems may impair the quality of survival, it is important to consider whether they could be reduced or prevented by schemes of counselling and rehabilitation, and the specific methods that might be used. However, few centres have yet implemented such schemes, and there is little literature to guide those who wish to improve the quality of their aftercare. So it was with eager anticipation that I approached this book, particularly as the editors stated on the front cover that it would provide detailed descriptions of the " various types of techniques which are used by rehabilitation counsellors, psychologists and others concerned with the rehabilitation of the individual with cancer ".

When I scanned the list of 7 contributors I became less optimistic. Only one of them appeared to be primarily concerned with cancer patients. My doubts grew on reading the first chapter " Cancer and Rehabilitation ". It is far too general and of little value to all except the " lay-counsellor ".

Although the second chapter discusses related " Medical and Psychological Problems " and provides a few useful pointers, it suffers from being too superficial and from its reliance on data published in 1959. This failure to acknowledge or discuss important recent developments in cancer counselling and rehabilitation is even more apparent in Chapters 3 and 4.

Chapter 3 on " Psychotherapeutic Work with the Cancer Patient " might have been taken straight out of a textbook on psychotherapy. It is full of jargon and contains virtually no discussion of how these very time-consuming methods may be applied to patients with cancer. Similarly, the next chapter on " Ileostomy Management " refers almost exclusively to " ulcerative colitis " sufferers. Hence, it concludes erroneously that patients who have anorectal cancer do not usually develop sexual problems after colostomy. The fifth and final chapter of " Case studies " fails to redeem the book, because it is equally superficial and unhelpful.

A further major criticism of this book is that it is repetitive. The nature of cancer is twice discussed at length, while the issue of whether to tell is tackled in 3 successive chapters, but never satisfactorily.

Overall, I got the impression of a book which was put together prematurely before adequate experience or awareness of other work had been gained by all but one of the contributors. This is most unfortunate, for it may simply confirm the prejudices of those who believe that the behavioural sciences have little to offer to cancer care. However, there is just a chance that it may encourage those already interested in this field to realise the urgent need for careful experimentation with different methods of counselling.

P. Maguire